# Ein neues Verständnis von Risikokommunikation in Public-Health-Notlagen

**DOI:** 10.1007/s00103-022-03529-8

**Published:** 2022-04-04

**Authors:** Petra Dickmann, Brigitte Strahwald

**Affiliations:** 1 Klinik für Anästhesiologie und Intensivmedizin – Public Health Hub, Universitätsklinikum Jena, Am Klinikum 1, 07747 Jena, Deutschland; 2 Pettenkofer School of Public Health, Institut für medizinische Informationsverarbeitung, Biometrie und Epidemiologie – IBE, Ludwig-Maximilians-Universität München, München, Deutschland

**Keywords:** Risikokommunikation, Krisenkommunikation, Public Health, Surveillance, Detektion, Response, Risk communication, Crisis communication, Public health, Surveillance, Detection, Response

## Abstract

Die Risikokommunikation öffentlicher Institutionen soll die Bevölkerung im Falle bestehender Risiken bei der Entscheidungsfindung unterstützen. In gesundheitlichen Notlagen wie der Coronavirus(SARS-CoV-2)-Pandemie spielt sie eine besonders wichtige Rolle. Bereits nach dem SARS-Ausbruch im Jahr 2003 hat die Weltgesundheitsorganisation (WHO) ihre Internationalen Gesundheitsvorschriften (IHR 2005) überarbeitet und gefordert, Risikokommunikation in allen Mitgliedsländern als einen Kernbereich in der Gesundheitspolitik zu etablieren. Während der gesundheitspolitische Akzent begrüßt wurde, konnten die Möglichkeiten der Risikokommunikation in diesem Bereich bisher nicht voll ausgeschöpft werden. Gründe sind u. a. Unstimmigkeiten im Begriffsverständnis der Risikokommunikation und die Vielzahl zur Verfügung stehender Methoden.

Der vorliegende Diskussionsartikel soll dazu beitragen, ein neues Verständnis von Risikokommunikation in Public-Health-Notlagen (Emergency Risk Communication – ERC) zu etablieren. Es wird vorgeschlagen, neben den Risiken die Chancen der Krise stärker einzubeziehen und Risikokommunikation noch mehr als einen kontinuierlichen Prozess zu begreifen, der an verschiedenen Stellen optimierbar ist. Der Earlier-Faster-Smoother-Smarter-Ansatz und hierbei insbesondere die frühere Erkennung von Gesundheitsgefahren (Earlier) könnten das Management von Public-Health-Notlagen zukünftig unterstützen.

## Abstract (en)

Risk communication of public institutions should support the population in the decision-making process in the event of existing risks. It plays a particularly important role in health emergencies such as the SARS-CoV‑2 pandemic. After the SARS outbreak in 2003, the World Health Organization (WHO) revised its International Health Regulations (IHR 2005) and called for risk communication to be established as a core area of health policy in all member countries. While the emphasis on health policy was welcomed, the potential for risk communication in this area has not yet been fully exploited. Reasons include discrepancies in the understanding of risk communication and the large number of available methods.

This discussion article is intended to help establish a new understanding of risk communication in public health emergencies (emergency risk communication – ERC). It is suggested that, in addition to the risks, the opportunities of the crisis should be included more and that risk communication should be understood more as a continuous process that can be optimized at various points. The “Earlier-Faster-Smoother-Smarter” approach and in particular the earlier detection of health risks (“Earlier”) could support the management of public health emergencies in the future.

## Hintergrund

Risikokommunikation ist ein weiter Begriff, der in den letzten Jahren eine Reihe von grundlegenden konzeptionellen Veränderungen erfahren hat. Im Rahmen dieses Beitrags konzentrieren wir uns auf die Risikokommunikation im Bereich von Public-Health-Notlagen.

Public Health als Wissenschaftsdisziplin und Praxis hat die Gesundheit der Bevölkerung im Blick sowie die Frage, wie diese – positiv oder negativ – beeinflusst wird und verbessert werden kann. Es geht dabei nicht nur um individuelles Verhalten, sondern auch und vor allem um die Verhältnisse und Rahmenbedingungen, in denen Menschen gesund leben können: also um Gesundheits‑, Bildungs- und Wirtschaftssysteme ebenso wie um politische Systeme, Strukturen von Beteiligungen und vieles mehr. Nachdenken und Entscheiden in Public Health kann daher nicht auf Gesundheitsfragen reduziert werden, sondern berührt genuin politische Felder und öffentliche Bereiche und muss gesellschaftlich ausgehandelt werden. Die Einführung des Rauchverbots im öffentlichen Raum ist ein Beispiel, das zeigt, wie Public Health zu gesellschaftlichen Veränderungen beiträgt und neue Normalitäten schafft.

Die inter- und transdisziplinäre Public-Health-Perspektive erlaubt einen reflektierten Blick auf die verschiedenen Gegenstandsbereiche. Sie eröffnet nicht nur Lösungen, sondern macht auch sichtbar, wie Entscheidungen zustande kommen und verhandelt werden. Sie richtet den Blick auf die Systeme, Mechanismen und Bedingungen von Entscheidungen und geht damit über das „*Was* ist gesund?“ hinaus zum „*Wie* ist Gesundheit möglich?“.

Diese reflektierende Eigenschaft teilt Public Health mit der Theorie und Praxis der Risikokommunikation. Risikokommunikation hat eine Reihe von „Moden“, Denkschulen und Praktiken durchlaufen und findet zu verschiedenen Anlässen mit unterschiedlichen Methoden statt. Ironischerweise ist der Begriff „Risikokommunikation“ dabei selbst häufig eine Quelle für Missverständnisse in der Kommunikation. Dies liegt an der oft unklaren Differenzierung von Risiko- und Krisenkommunikation, an der ebenso unklaren Definition der zugrunde liegenden Begriffe Risiko, Kommunikation und Public Health sowie an dem heterogenen Verständnis der Rolle im Risiko- bzw. Krisenmanagement und an der konkreten Umsetzung in der Praxis.

Gleichzeitig ist Risikokommunikation in den letzten Jahren ein eigener Forschungsschwerpunkt geworden, der verschiedene Theorien, Modelle und Methoden umfasst [1]. In der Regel erfolgt diese Forschung aber relativ strikt aus der Sicht einer einzelnen Fachdisziplin. Auch die Public-Health-Forschung ist in dem Bereich wenig inter- und transdisziplinär. Dadurch werden potenziell wertvolle und wichtige konzeptionelle Ideen, Denk- und Lösungsansätze nicht ausgeschöpft.

In diesem Diskussionsbeitrag sollen zunächst die Begriffe und Konzepte im Themenbereich Risikokommunikation in Public Health erläutert werden. Daraufhin wird begründet, warum positive Botschaften (Chancen) stärker bei der Risikokommunikation aufgegriffen werden sollten. Und es wird aufgezeigt, dass die Risikokommunikation in einer Public-Health-Notlage als ein kontinuierlicher Prozess verstanden werden kann – ein Konzept, das verschiedene Ansatzmöglichkeiten für Interventionen bietet und das Management von Public-Health-Notlagen zukünftig unterstützen kann.

## Begriffe und Konzepte in der Public-Health-Risikokommunikation

### Risikokommunikation

Sehr vereinfacht geht es bei der Risikokommunikation in Public Health um die Kommunikation von Wahrscheinlichkeiten, mit denen Gesundheitsgefahren, die sich aus bestimmten Verhaltensweisen und/oder Verhältnissen ergeben, negative Konsequenzen für Bevölkerungsgruppen nach sich ziehen können. Ein Beispiel ist die Kommunikation über die Gefahren von Feinstaub. Eine hohe Feinstaubbelastung in der Luft erhöht unter anderem die Wahrscheinlichkeit, im Verlauf des Lebens an Asthma zu erkranken. Diese Wahrscheinlichkeit wurde in der Regel aus Bevölkerungsdaten ermittelt und ist – je nach Datenlage – unterschiedlich präzise und verlässlich.

Aus Public-Health-Perspektive zielt die Risikokommunikation zum einen darauf ab, die negativen Konsequenzen für eine betroffene Population, beispielsweise in einem Wohnbereich an der Autobahn, zu verringern. Diese Kommunikation ist vor allem für politische und gesamtgesellschaftliche Entscheidungen relevant. Zum anderen werden Einzelpersonen informiert, wie sie ihr individuelles Risiko verringern oder sogar eliminieren können.

Um die Botschaften verstehen und danach handeln zu können, ist aufseiten der Informationsempfänger oft ein hohes Maß an Gesundheitskompetenz sowie an solider Daten- bzw. statistischer Kompetenz erforderlich. Zahlreiche Untersuchungen haben gezeigt, dass diese Kompetenzen nicht vorausgesetzt werden können [2, 3]. Ein weiterer einflussreicher Faktor in diesem Zusammenhang ist die Risikowahrnehmung (*Risk Perception*), sowohl individuell als auch auf Bevölkerungsebene [4, 5]. Diese Wahrnehmung wird oft nicht durch die Daten und Wahrscheinlichkeiten geprägt, sondern durch eine Vielzahl anderer Einflüsse wie Werte, Erfahrungen und Emotionen. So entstehen „gefühlte Risiken“, die Entscheidungen oft maßgeblicher beeinflussen als fakten- und datenbasierte Informationen.

### Krisenkommunikation

Krisenkommunikation im engeren Sinn bezieht sich in Public Health auf eine gesundheitsgefährdende Situation, die bereits eingetreten ist (Tab. 1). In diesem Beitrag wird sie auch als „Risikokommunikation in Public-Health-Notlagen“ (Emergency Risk Communication – ERC) bezeichnet, ein Begriff der von der Weltgesundheitsorganisation (WHO) verwendet wird. Im Kern geht es darum, Schäden zu begrenzen und weitere abzuwenden. Ein aktuelles Beispiel ist die Coronapandemie, allerdings hat diese Krise einen besonderen Charakter. Als erste schwere Pandemie im Zeitalter der sozialen Medien hat sie die Krisenkommunikation vor neue Herausforderungen gestellt, aber auch neue Chancen eröffnet. Nüchtern betrachtet waren die Vorbereitungen auf diese Krise in Hinblick auf die Krisenkommunikation allerdings nicht ausreichend, wie erste Zwischenbilanzen zeigen [6, 7].

**Table 1:** 

	Anlass
*Risikokommunikation*	Gesundheitsgefährdende Verhaltensweisen und/oder Verhältnisse
*Krisenkommunikation* (Synonym: Risikokommunikation in Public-Health-Notlagen)	Plötzliches, gesundheitsgefährdendes Ereignis

### Risikokommunikation und Gesundheitspolitik

Die WHO räumt der Risikokommunikation in Public-Health-Notlagen einen hohen Stellenwert ein. Nach dem ersten Coronavirusausbruch 2003 wurden die Internationalen Gesundheitsvorschriften (IHR 2005) überarbeitet; diese stufen die Risikokommunikation als eine von 13 Kernkompetenzen (*Core Capacities*) der Mitgliedstaaten ein [8]. Dem zugrunde liegt ein völlig neues Verständnis von Risikokommunikation: „Früher wurde Risikokommunikation in erster Linie als Übermittlung von Informationen an die Öffentlichkeit über Gesundheitsrisiken und -ereignisse betrachtet, … sowie als Anleitung zu Verhaltensänderungen, um dieses Risiko zu reduzieren. Mit der Entwicklung sozialwissenschaftlicher Erkenntnisse sowie neuer Kommunikations- und Medientechnologien und -praktiken im 21. Jahrhundert hat sich die Denkweise in diesem Bereich dramatisch verändert“ (eigene Übersetzung; [9]).

Gleichzeitig rückt die WHO das Verständnis und die Praxis von Risikokommunikation in den Bereich der politischen Steuerung (*Governance*). Aus der zuvor eher begleitend gedachten Risikokommunikation wird ein essenzieller Teil von Krisenvorsorge und -management, den jedes Mitgliedsland der Vereinten Nationen vorhalten muss. Risikokommunikation in Public-Health-Notlagen ist damit völkerrechtlich verankert und gehört mit zu den zentralen Elementen der Vorbereitung, Vorbeugung und Bewältigung von Gesundheitskrisen.

Die WHO hat zur Stärkung des Öffentlichen Gesundheitsdienstes eine Reihe von Werkzeugen und evidenzbasierten Empfehlungen veröffentlicht, mit denen Public-Health-Institutionen in die Lage versetzt werden sollen, Risikokommunikation in Notlagen und im Alltag pragmatisch und gut umzusetzen [10]. Das zugrunde liegende Konzept legt den Schwerpunkt auf eine gemeinsame Risikobewertung und -kommunikation durch Gesundheitsbehörden, Interessenvertretungen und Bevölkerungsgruppen. Aus den überholten unilateralen Kommunikationswegen sollen Interaktionen werden, wie auch die Definition der WHO zeigt. Risikokommunikation ist demnach: „… der Austausch von Informationen, Ratschlägen und Meinungen in Echtzeit zwischen Experten oder Behörden und Menschen, die einer Bedrohung (Gefahr) für ihr Überleben, ihre Gesundheit oder ihr wirtschaftliches oder soziales Wohlergehen ausgesetzt sind. Letztlich geht es darum, dass alle gefährdeten Personen in der Lage sind, fundierte Entscheidungen zu treffen, um die Auswirkungen der Bedrohung (Gefahr), z. B. eines Krankheitsausbruchs, zu mindern und Schutz- und Präventionsmaßnahmen zu ergreifen“ (eigene Übersetzung; [11]). Damit wird der Begriff der Risikokommunikation erweitert und kann zusätzlich als Plattform und Diskursraum verstanden werden, in dem diese Interaktionen stattfinden können.

**Figure 1:**
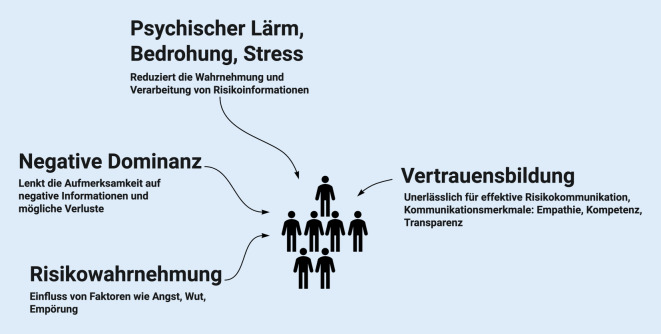

Einige Modelle und Theorien für Risikokommunikation sind hilfreich, um typische Einflüsse auf die Wirksamkeit und Störgrößen abzuschätzen und einzuordnen (Abb. 1). In der aktuellen Übersicht von Loss et al. wird beschrieben, dass Bedrohung und Stress die Wahrnehmung und das Verständnis der Risikoinformationen beeinträchtigen (*Theorie des psychischen Lärms*; [6]). Zudem werden in Krisen eher negative Informationen und Ergebnisse aufgenommen als Chancen, die häufig aber gar nicht kommuniziert werden (*Modell der negativen Dominanz*). Und wie bereits erläutert, stimmt die Wahrnehmung von Risiken häufig nicht mit dem objektiven Schadenspotenzial überein (*Theorie der Risikowahrnehmung*). Vor allem emotionale Aspekte wie Angst, Wut oder das Gefühl der Bedrohung beeinflussen die Wahrnehmung. Umgekehrt ist ein zentraler Anspruch an gute Risikokommunikation, dass Vertrauen aufgebaut, erhalten oder gar verstärkt wird (*Theorie der Vertrauensbestimmung*).

Für die Praxis der Risikokommunikation in Public-Health-Notlagen ist es eine enorme Herausforderung, all diese Einflüsse zu berücksichtigen, denn bei allen Antworten und Lösungen müssen die äußerst heterogenen Bedarfe und Voraussetzungen innerhalb der Bevölkerung, aber auch bei Institutionen, Behörden und in der Politik berücksichtigt werden. Ein rein kognitives Vorgehen bei der Risikokommunikation ist dabei ebenso wenig ausreichend wie ein rein emotionales; vielmehr gilt es, eine adäquate, zielgruppenspezifische Lösung zu finden.

## Neue Herangehensweisen an die Risikokommunikation in Public-Health-Notlagen

Um die bestehenden Ansätze der Risikokommunikation zu verbessern und vor allem mit Blick auf den *Governance*-*Ansatz*, werden im Folgenden 2 neue Herangehensweisen zur Diskussion gestellt:

- Schwerpunktsetzung auf positive Botschaften (Chancen) und Entwicklung einer „Risiko- und Chancenkommunikation“,
- erweitertes Verständnis von Risikokommunikation als Prozess.

Um diese beiden Ansätze zu erläutern, gehen wir nochmals genauer auf die Begriffe „Kommunikation“ und „Risiko“ im Zusammenhang mit Public Health ein. Sie befinden sich im Wandel, während eine entsprechende Anpassung der Risikokommunikation in der Praxis nur teilweise gelingt.

### Kommunikation

Die Kommunikation im Bereich Public Health soll nach einem neueren Verständnis explizit nicht mehr auf Modellen einseitiger Top-down-Botschaften beruhen, sondern idealerweise partizipativ geplant, entwickelt und umgesetzt werden. Basierend auf dem Vorschlag der WHO zum *Governance*-Ansatz von Risikokommunikation sollten die Interaktionen mit verschiedenen Bevölkerungsgruppen und Multiplikatoren verstärkt werden, um sowohl die Inhalte als auch die Methode von Risikokommunikation stärker gemeinsam zu verhandeln. Dieser Anspruch wird jedoch häufig nicht erfüllt, was sich gerade in der Coronapandemie gezeigt hat [12].

In einem aktuellen Scoping-Review zur Public-Health-Risikokommunikation wird konstatiert, dass während der Pandemie der Fokus auf technischen Informationskanälen lag [13]. Zudem gab es eine mangelnde Diversität, sowohl was Bevölkerungsgruppen angeht wie auch verschiedene Kommunikationsarten [13]. Zu ähnlichen Schlüssen kommt auch eine weitere Übersichtsarbeit, die die Strategien zur Risikokommunikation und Einbindung der Bevölkerung (*Risk Communication and Community Engagement,* RCCE) in gefährdeten Ländern und Bevölkerungsgruppen in den frühen Pandemiephasen untersucht [7]. Demnach sind den Top-down-Modellen des RCCE klare Grenzen gesetzt, während die Aufforderung zur „Einbindung der Bevölkerung“ neu konzeptionalisiert und praktiziert werden muss, um möglichst viele Personen und Gruppen in einem umfassenden Sinn einzubeziehen. Dies gelang nicht in ausreichendem Maß.

Ein weiterer Anspruch ist, dass die Kommunikation im Bereich Public Health neben dem kognitiven Vorgehen auch emotionale Aspekte, Werte und Erfahrungen berücksichtigen muss, die für die Risikowahrnehmung oft entscheidend sind. Dies kollidiert jedoch mit dem in westlichen Ländern dominierenden rationalen Konzept von Gesundheit und der entsprechenden Wahl der Kommunikationsformen und -kanäle. Die häufig empfohlenen Faktenboxen sind ein Beispiel dafür. Sie basieren zumindest teilweise auf der Annahme, dass Menschen sich, wenn sie die Statistik ihrer Gesundheitsrisiken besser verstehen, risikoärmer bzw. gesundheitsbewusster verhalten. Faktenboxen liefern die entsprechenden evidenzbasierten Informationen mithilfe von Text und Datenvisualisierung [14, 15]. Die Strategie basiert auf den psychologischen Forschungen zu individuellen Entscheidungsfindungen. Auch wenn dieses Vorgehen ein sinnvoller Ansatz ist, bleiben Lücken in der Kommunikation, die derzeit noch nicht geschlossen werden.

Aus diesem Verständnis heraus ist es auch schwierig, die Rolle und die Dynamik der Kommunikation in sozialen Medien oder speziell von *Influencern* zu verstehen und positiv zu nutzen. Die unregulierte und nichtredigierte Kommunikation auf Social-Media-Plattformen wird von Public-Health-Verantwortlichen zumeist negativ aufgefasst und als „Infodemie“ beschrieben, die – ähnlich wie die verursachende Viruserkrankung – besser eingedämmt werden sollte [16–18]. Dabei können Gerüchte und schnelle Meldungen über Social-Media-Kanäle auch eine wichtige Rückmeldung für die Risikobewertung und die -kommunikation sein und zu einer Adjustierung von Handlungsstrategien und Maßnahmen führen [19]. Die „Kontagiosität von Narrativen“ ist ein wichtiger Forschungszweig der Sozial‑, Geistes- und Datenwissenschaften, aus dem interessante Erkenntnisse für eine neue Theorie und Praxis der Risikokommunikation in Public-Health-Notlagen gewonnen werden können [20, 21].

Um die von der WHO geforderte Teilnahme- und Teilhabe der Bevölkerung an den Zielen und Methoden der Risikokommunikation in Public-Health-Notlagen zu realisieren, sind neue und/oder ergänzende Ansätze hilfreich. Neben dem fakten- und datengetriebenen Kommunikationsansatz sollten partizipative und fluide Lösungen für die weiteren Kommunikationsbedarfe gefunden werden.

### Risiko

Der Begriff Risiko wird in den verschiedenen Fachbereichen unterschiedlich definiert. Im Allgemeinen wird Risiko als Produkt aus dem (vermuteten) Schadensausmaß und der (berechneten) Eintrittswahrscheinlichkeit verstanden. Dieses mathematische Verständnis von Risiko wird als eher rückwärtsgewandtes Verfahren kritisch diskutiert, da es wenig Spielraum lässt, neue Risiken und das Zusammenwirken verschiedener, neuer Risiken zu verstehen. Künftige Entwicklungen lassen sich damit nur unzureichend erfassen.

Sandman prägte die Formel vom Risiko als Summe aus Gefahr und Empörung („hazard plus outrage“), wobei die erwähnten emotionalen Aspekte der Risikowahrnehmung integriert wurden [22]. Er unterscheidet sinnvollerweise zwischen „Gefahr“ und „Risiko“, allerdings lassen sich künftige Entwicklungen auch mit diesem Ansatz nur unzureichend erfassen.

In der Organisationslehre und dem Risikomanagement wird Risiko dagegen als Abweichung von den Zielen eines Unternehmens oder einer Organisation verstanden. Diese Betrachtungsweise erlaubt ein anderes Nachdenken über Risiken, das der Dynamik von Risiken in der Realität besser gerecht werden kann. Interessanterweise wird hier auch der Chancenbegriff integriert, der in den traditionelleren Modellen regelhaft fehlt.

### Wandel zur „Risiko- und Chancenkommunikation“

Die Entwicklungen im Verständnis der grundlegenden Begriffe ermöglichen es, auch den Begriff der Risikokommunikation in Public-Health-Notlagen neu zu denken und den Fokus stärker in Richtung einer Kommunikation von Chancen zu verschieben.

Das Aufgreifen des Chancenbegriffs ist dabei eine gezielte Strategie. Mit dem Wandel zur Risiko- und Chancenkommunikation könnten Droh- und Furchtbotschaften mit ihren problematischen Auswirkungen einfacher und nachhaltiger vermieden werden. Wie oben beschrieben, werden in Krisen negative Informationen eher wahrgenommen und im Gedächtnis behalten [23, 24].

Die bisherige Fokussierung auf Risiken ist umso erstaunlicher, als sich gezeigt hat, dass eine permanente Alarmierung negative Folgen haben kann, wenn auch auf individuell unterschiedliche Weise. Neben einer zunehmenden Reaktanz und einer Bagatellisierung des Risikos können umgekehrt panikartige und damit riskante Reaktionen resultieren. Zudem ist allgemein anerkannt, dass idealerweise konkrete Möglichkeiten aufgezeigt und kommuniziert werden sollen, damit alle aktiv an Lösungen mitwirken können [10, 25, 26].

Die Kommunikation von Chancen bedeutet nicht, dass Risiken verschwiegen oder falsch dargestellt werden sollen; ebenso wenig geht es um eine unkritische Reduktion auf positive Botschaften. Vielmehr soll auf eine Balance geachtet werden, die aber bewusst Wege zur Selbstermächtigung (*Empowerment*) in Notlagen aufzeigt. Eine Impfkampagne kann beispielsweise die individuellen und gesellschaftlichen Chancen auf den gesundheitlichen Schutz, einen Zugewinn an Freiheiten und die Möglichkeit, selbst darüber mitzuentscheiden, vermitteln – oder aber die individuellen und gesellschaftlichen Risiken betonen und die Mitwirkung durch Sanktionen forcieren. Studien legen nahe, dass schon die Reihenfolge der Kommunikation von positiven und negativen Botschaften einen Einfluss auf die Wahrnehmung hat ebenso die Wiederholung der Inhalte [27].

Ob und wie die Verschiebung des Fokus von der Risiko- zur Chancenkommunikation in Public-Health-Notlagen konkret wirkt, müssen entsprechende zukünftige Studien zeigen.

### Risikokommunikation als Prozess: das „Earlier-Faster-Smoother-Smarter-Konzept“

Das veränderte Verständnis von Kommunikation und Risiken ermöglicht auch ein neues Verständnis von Risikokommunikation als kontinuierlichen Prozess mit mehreren Stellschrauben im Sinne des Governance-Ansatzes. Jedes gesundheitsbezogene Ereignis hat eine Dynamik, auf die die Interventionen abgestimmt werden können.

**Figure 2:**
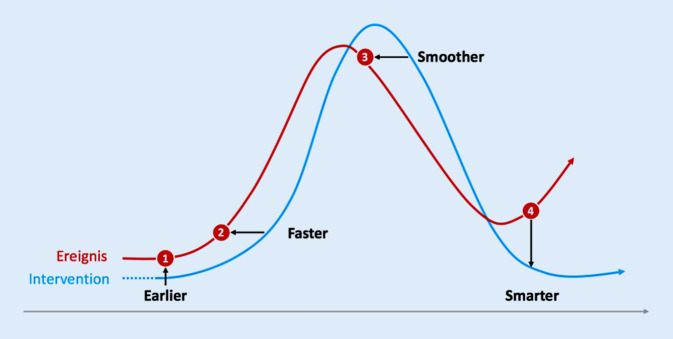

Die sich ergebende Interventionsdynamik wird in Abb. 2 dargestellt. Durch ein neues Verständnis von Risikokommunikation könnte die Interventionsdynamik an 4 Stellen optimiert werden [28]:

1. EARLIER: Risikokommunikation soll Methoden entwickeln und Aktivitäten umsetzen, die es ermöglichen, *näher* an den „Communitys“ zu sein und die vereinzelten Ausbrüche, die zunächst als unklare Geschehnisse auftreten, *früher *aufzunehmen („das Ohr am Boden haben“).
2. FASTER: Außerdem soll Risikokommunikation die Flexibilität ermöglichen, *schneller* auf Abweichungen zu reagieren.
3. SMOOTHER: Risikokommunikation soll es ermöglichen, die Krise *koordinierter* zu gestalten.
4. SMARTER: Als wichtiger Bereich im Management von Krisen ist das Lernen aus vergangenen Ereignissen ein zentrales Element, um tatsächlich in Zukunft besser auf Krisen vorbereitet zu sein. Risikokommunikation soll es ermöglichen, Prozesse zu reflektieren und Verbesserungen nachhaltig umzusetzen. Ebenso können aus den Erfahrungen Risikofaktoren abgeleitet werden, die dann dazu führen kann, Krisen zu verhindern und sie früher (Earlier) zu erkennen.

### EARLIER: Gesundheitsgefahren früher entdecken

Ein neues Verständnis von Risiko, Kommunikation und Risikokommunikation ändert auch die Arbeits- und Aufgabenbereiche von Risikokommunikation in Public-Health-Notlagen [28, 29]. Der Wandel hin zum *Governance*-Konzept wird hier am Beispiel von Earlier beschrieben:

Im Bereich der Früherkennung von Gesundheitsgefahren mit Notlagenpotenzial spielt Risikokommunikation traditionell eine große Rolle. Das Earlier-Konzept soll es ermöglichen, Gesundheitsgefahren früher zu erkennen. Dazu ist es nötig, die Kernaufgaben von Risikokommunikation (Information – Kommunikation – Koordination) so anzuwenden, dass das „Ohr näher am Boden“ ist, Informationen also frühzeitig aufgenommen werden. Die psychologische Forschung weist auf die Bedeutung eines Monitorings von Verhalten hin [30]. Mithilfe von Surveys werden die Einstellung, das Wissen und die Wahrnehmung der Befragten erfasst. Außerdem kann die Analyse von Gerüchten und Nachrichten in sozialen Medien dabei helfen, Geschehnisse frühzeitig zu erkennen [31, 32]. Die Analyse von Verhalten, Wahrnehmungen und insbesondere informellen, schnellen Publikationen könnte in etablierte Surveillance-Strukturen integriert werden, die vorwiegend auf die biomedizinischen Parameter ausgerichtet sind [33]. Außerdem gibt es im Bereich der Sozialwissenschaften interessante Entwicklungen aus sog. Soziallaboren (Social Laboratories), die die Intelligenz und Vernetzung von Gruppen nutzen, um Entwicklungen früher zu erkennen.

Diese Methoden haben bei der Früherkennung von Infektionserkrankungen eine besondere Bedeutung, denn je früher diese erkannt werden, desto schneller kann die Weiterverbreitung gestoppt werden [34, 35]. Die Internationalen Gesundheitsvorschriften (IHR 2005) tragen zur Entwicklung von Methoden bei, die es erlauben, Ausbrüche früher zu erkennen und dabei auf die Ortskenntnis und Aufmerksamkeit der Menschen vor Ort zu setzen [36]. Mithilfe der Soziallabormethode konnten bei der Bekämpfung des Ebolaausbruchs 2014/2015 in Westafrika tatsächlich neue Wege zum Infektionsmanagement gefunden werden [37].

### Partizipation der Menschen vor Ort

Um Gesundheitsgefahren, wie etwa Ausbrüche von Infektionserkrankungen, frühzeitig erkennen zu können, ist es sinnvoll, bei den Menschen vor Ort (innerhalb der Communitys) Abweichungen von der gewohnten Situation zu registrieren, die auf erste Erkrankungsfälle hindeuten. In einem nächsten Schritt müssen die Menschen (Laien, Haus- oder auch Tierärzte) wissen, wie sie die Gesundheitsämter erreichen können, um entsprechende Beobachtungen zu teilen. Die Gesundheitsämter müssen diese Informationen aufnehmen, bewerten und mit regionalen Kollegen teilen und gleichzeitig erkennen, dass es ein Ausbruch ist, und angemessene Interventionen zur Eindämmung einleiten. Dieses Konzept zur Früherkennung von Krankheitsausbrüchen wird aktuell für Public-Health-Notlagen aufgearbeitet [34, 36].

Eine zentrale Rolle bei der früheren Erkennung spielen also die Menschen vor Ort. Sie können und sollten stärker in die Infektionserkennung und die Infektionskontrolle einbezogen werden. Viele gute, pragmatische und nachhaltige Vorschläge kommen dabei direkt aus den betroffenen Gruppen.

Wie wichtig der Einbezug dieser Gruppen ist, zeigen auch Untersuchungen während dieser Pandemie. So wurde am Beispiel einer orthodoxen jüdischen Gemeinschaft demonstriert, wie ein Verständnis des Wertesystems einer Gemeinschaft dazu beitragen kann, eine Gesundheitskrise gemeinschaftlich zu meistern [38]. Eine aktuelle Untersuchung zur Risikokommunikation der Quarantäne für ein gesamtes Dorf zu Beginn der Coronapandemie zeigt die Bedeutung von sozialen Zusammenhängen auf, die sowohl für die Virusverbreitung relevant sind als auch die Kommunikation zwischen Bevölkerung und Gesundheitsämtern erleichtern – und von der Public-Health-Risikokommunikation stärker genutzt werden sollte [39]. Zudem bieten die neuen technischen Möglichkeiten des 21. Jahrhunderts eine Reihe von konzeptionellen Veränderungen in der Partizipation an, die noch weiter exploriert werden sollten.

## Fazit und Ausblick

Risikokommunikation hat in Public-Health-Notlagen ein besonderes Potenzial, das in der Praxis noch nicht vollständig ausgeschöpft wird. Dieser Beitrag beschreibt die aktuellen konzeptionellen Entwicklungen der Risikokommunikation von einer eher technisch-disziplinären Tätigkeit zu einem wichtigen Instrument der Gesundheitspolitik. Die Betonung von Chancen und ein Verständnis von Risikokommunikation als Prozess bieten zusammen mit Partizipation neue Möglichkeiten für eine gelingende Risikokommunikation.
